# Dr. Frank P. Bingham

**Published:** 1901-09

**Authors:** 


Dr. Frank P. Bingham, of Buffalo, was crossing car tracks at Main and Summer streets on his bicycle on August 2, 1901, when he was struck by a street car and so severely injured that he died in a few minutes. He was a son of Charles W. Bingham, of No. 525 Niagara Street. He was born in Pennsylvania and received his education in the University of Vermont and the University of Buffalo, graduating from the latter in 1899. He was a nephew of George C. Bingham, vice-president and treasurer of the American Agricultural & Chemical Company. He had lived in Buffalo for nine years and two years he was physician at the Children's Hospital at Athol Springs. He was held in high esteem by all who knew him, especially in the profession which he had chosen, and in which he bid fair to become highly successful. His age was 28 years.

